# Cardiac ryanodine receptor activation by a high Ca^2+^ store load is reversed in a reducing cytoplasmic redox environment

**DOI:** 10.1242/jcs.156760

**Published:** 2014-10-15

**Authors:** Amy D. Hanna, Alex Lam, Chris Thekkedam, Esther M. Gallant, Nicole A. Beard, Angela F. Dulhunty

**Affiliations:** 1 John Curtin School of Medical Research, Australian National University, Canberra, ACT 6200, Australia; 2 Centre for Research in Therapeutic Solutions, University of Canberra, Bruce, ACT 2617, Australia

**Keywords:** Cardiac ryanodine receptor, Redox potential, Sarcoplasmic reticulum, Luminal Ca^2+^ sensitivity

## Abstract

Here, we report the impact of redox potential on isolated cardiac ryanodine receptor (RyR2) channel activity and its response to physiological changes in luminal [Ca^2+^]. Basal leak from the sarcoplasmic reticulum is required for normal Ca^2+^ handling, but excess diastolic Ca^2+^ leak attributed to oxidative stress is thought to lower the threshold of RyR2 for spontaneous sarcoplasmic reticulum Ca^2+^ release, thus inducing arrhythmia in pathological situations. Therefore, we examined the RyR2 response to luminal [Ca^2+^] under reducing or oxidising cytoplasmic redox conditions. Unexpectedly, as luminal [Ca^2+^] increased from 0.1 to 1.5 mM, RyR2 activity declined when pretreated with cytoplasmic 1 mM DTT or buffered with GSH∶GSSG to a normal reduced cytoplasmic redox potential (−220 mV). Conversely, with 20 µM cytoplasmic 4,4′-DTDP or buffering of the redox potential to an oxidising value (−180 mV), RyR2 activity increased with increasing luminal [Ca^2+^]. The luminal redox potential was constant at −180 mV in each case. These responses to luminal [Ca^2+^] were maintained with cytoplasmic 2 mM Na_2_ATP or 5 mM MgATP (1 mM free Mg^2+^). Overall, the results suggest that the redox potential in the RyR2 junctional microdomain is normally more oxidised than that of the bulk cytoplasm.

## INTRODUCTION

There is much evidence linking excess diastolic Ca^2+^ release with the onset of delayed afterdepolarisations (DADs) and arrhythmia in a number of pathological conditions (Fauconnier et al., 2011; Marks, 2013; Pogwizd and Bers, 2004; Shannon et al., 2002). This excessive Ca^2+^ leak has been linked to enhanced β adrenergic activity that leads to hyperphosphorylation of RyR2 channels and to oxidative stress. It has been suggested that redox modification of RyR2, caused by the production of excess reactive oxygen species (ROS), could contribute to enhanced RyR2 sensitivity to luminal [Ca^2+^] in canine heart failure (Belevych et al., 2009; Shan et al., 2012; Terentyev et al., 2008). This was elegantly illustrated by the finding of a significantly greater open probability of RyR2 channels from failing hearts than in channels from healthy hearts when the luminal [Ca^2+^] was 0.02 mM. The open probability of the channels from failing hearts was reduced to values found in healthy hearts by the addition of dithiothreitol (DTT). By contrast, the open probability of RyR2 channels from healthy heart tissue was increased to levels seen in RyR2 from failing hearts by the addition of 4,4′-DTDP (Terentyev et al., 2008).

Given the importance of oxidation for the response of RyR2 channels to changes in luminal [Ca^2+^] in heart failure, we have, for the first time, explored the response of healthy RyR2 channels to physiological changes in luminal [Ca^2+^] over a range of cytoplasmic redox potentials. Extreme reducing or oxidising cytosolic redox potentials were set by the inclusion of 1 mM DTT or 20 µM 4,4′-DTDP, respectively, in the cytoplasmic solution bathing artificial planar bilayers. In addition, the luminal and cytosolic redox potentials were buffered to levels within a cellular range by using the major intracellular redox buffer – the GSH∶GSSG system (Hwang et al., 1992). The luminal redox potential was set at an oxidised level of −180 mV, and the cytoplasmic potential varied between an oxidised potential of −180 mV and a more reduced −220 mV (Feng et al., 2000; Feng and Pessah, 2002; Jalilian et al., 2008).

We have also explored the effect of buffered redox potential on the response of RyR2 to changes in luminal [Ca^2+^] in the presence of Mg^2+^ and/or ATP. Both ATP and Mg^2+^ are powerful endogenous ligands that regulate RyR2 activity during diastole. It was recently reported that RyR2 channels in bilayers are unresponsive to changes in luminal [Ca^2+^] in the presence of 1 mM free cytosolic Mg^2+^, which approximates the *in vivo* cytosolic [Mg^2+^] (Chen et al., 2013). This finding is at odds with experiments in intact cells, where there is a clear increase in Ca^2+^ efflux from the sarcoplasmic reticulum as Ca^2+^ is replenished during diastole (Lukyanenko et al., 1996; Lukyanenko et al., 2001; Satoh et al., 1997; Shannon et al., 2003).

We report a robust redox-dependent response of RyR2 to changes in luminal [Ca^2+^] within the physiological range of 0.1 to 1.5 mM, and this response is maintained in the presence of Mg^2+^ and ATP in GSH∶GSSG redox-buffered solutions. The usual increase in RyR2 activity with increasing luminal [Ca^2+^] in the absence of added redox reagents or when the cytoplasmic redox potential was oxidising was seen. Surprisingly, when the cytoplasmic redox potential was relatively reduced, as it is assumed to be in healthy myocytes, the open probability of RyR2 channels declined as luminal [Ca^2+^] increased. This is in marked contrast to myocyte studies in which Ca^2+^ leak through RyR2 increases as sarcoplasmic reticulum Ca^2+^ load increases (Shannon et al., 2002). We suggest that the difference between the bilayer and whole cell studies is that redox potential within the microdomain of the dyad junction in not as reduced as that of the bulk cytoplasm. A more oxidised redox potential might be maintained by local factors such as NADPH oxidase (NOX2) activity.

## RESULTS

In an artificial planar bilayer system, sarcoplasmic reticulum vesicles added to the cis solution incorporate into the bilayer with their cytoplasmic side facing the cis solution (Beard et al., 2002; Laver, 2005) and their luminal domain facing the trans solution. Therefore, the cis and trans solutions will be referred to as ‘cytoplasmic’ and ‘luminal’ solutions, respectively. The sequence of steps in all experiments after RyR2 incorporation into the bilayer was: (1) cytoplasmic [Ca^2+^] was reduced to 1 µM; (2) ATP or MgATP were added when required, then redox reagents were added; (3) luminal [Ca^2+^] was reduced to 0.1 mM and then increased in three steps to 1.5 mM. Channel activity was recorded for 4 min after each step, except with a luminal [Ca^2+^] of 0.1 mM, where recording was for 2 min in order to minimise calsequestrin (CSQ) dissociation (Beard et al., 2002).

### RyR2 response to luminal [Ca^2+^] in the absence of redox reagents

**Fig. 1.**
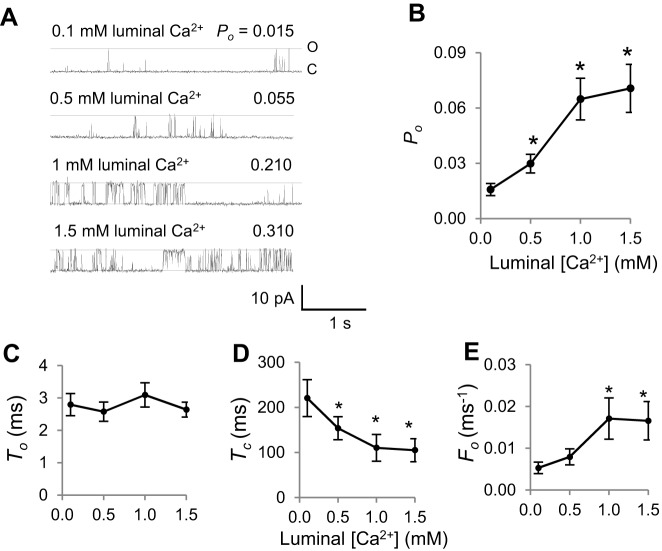
**Single RyR2 channel response to changes in luminal [Ca^2+^] in the absence of added redox reagents.** (A) Records of 3 s of single channel activity at +40 mV, where channel opening is upward from zero current (‘C’, continuous line) to maximum open conductance (‘O’, broken line) with a cytoplasmic [Ca^2+^] of 1.0 µM. Descending from the upper trace, the data show activity following luminal perfusion with a 0.1 mM Ca^2+^ solution followed by stepwise increase in luminal [Ca^2+^] – 0.5 mM, 1 mM and 1.5 mM. Open probability (*P*_o_) values for each recording are shown. (B–E) Mean data for *P*_o_, *n* = 17–24 (B); mean open time (*T*_o_), *n* = 12–15 (C); mean closed time (*T*_c_), *n* = 12–17, (D); and mean open frequency (*F*_o_), *n* = 12–17 (E). Error bars show ±s.e.m.; *P<0.05 (versus the value at 0.1 mM Ca^2+^).

The response of RyR2 channels to increasing luminal [Ca^2+^] has been determined in the absence of added redox reagents (Dulhunty et al., 2012; Qin et al., 2008; Sitsapesan and Williams, 1994; Terentyev et al., 2008). We repeated this experiment to establish that RyR2 channels responded in the usual way in the absence of added redox reagents. The mean open probability (*P*_o_) was 0.042±0.006 (±s.e.m.) at a luminal [Ca^2+^] of 1 mM and fell significantly to 0.016±0.003 when luminal [Ca^2+^] was lowered to 0.1 mM. There were significant increases in *P*_o_ as luminal [Ca^2+^] increased, to a mean *P*_o_ of 0.071±0.01 with 1.5 mM Ca^2+^ (Fig. 1A,B). The increase in *P*_o_ was due to a decrease in the mean closed time (*T*_c_), which, at 1 mM luminal Ca^2+^, was ∼50% of the value observed at 0.1 mM luminal Ca^2+^ (Fig. 1D). There was a parallel approximately threefold increase in the mean open frequency (*F*_o_), but no change in the mean open time (*T*_o_, Fig. 1C–E).

### The effect of 1 mM DTT in the cytoplasmic solution

**Fig. 2.**
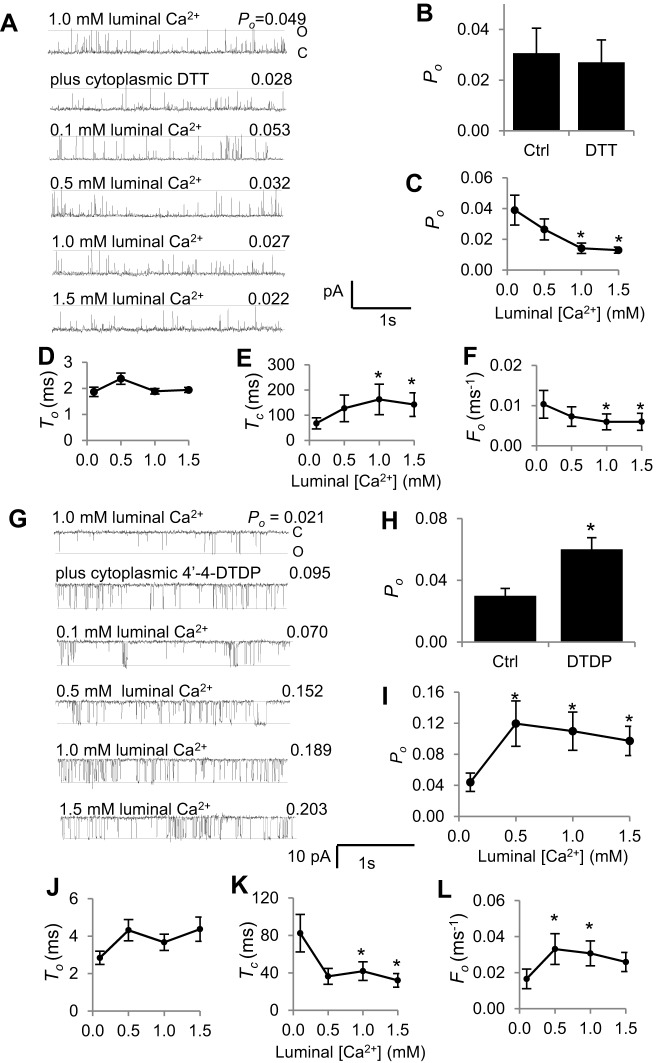
**Response of DTT-treated or 4,4′-DTDP-treated RyR2 channels to changes in luminal (trans) [Ca^2+^].** (A–F) Effects of DTT treatment. (A) 3 s of single channel activity at +40 mV, where channel opening is upward from zero current (‘C’, continuous line) to maximum open conductance (‘O’, broken line) with a cytoplasmic [Ca^2+^] of 1.0 µM. Open probability (*P*_o_) values for each recording are shown. Descending from the upper trace, the data show initial activity with 1.0 mM luminal Ca^2+^, activity after adding 1 mM DTT to the cytoplasmic solution, activity following luminal perfusion with a 0.1 mM Ca^2+^ solution, then activity when luminal [Ca^2+^] was increased stepwise to 0.5 mM, 1 mM and 1.5 mM. (B) Mean *P*_o_ before and after adding 1 mM DTT (*n* = 14). Ctrl, control. (C–F) Mean data (*n* = 6–14) for *P*_o_ (C); mean open time (*T*_o_) (D); mean closed time (*T*_c_) (E); and mean open frequency (*F*_o_) (F). (G–L) Effects of 4,4′-DTDP-treatment. (G) 3 s of single channel activity at −40 mV, where channel opening is downward from zero current (‘C’, continuous line) to maximum open conductance (‘O’, broken line) with a cytoplasmic [Ca^2+^] of 1.0 µM. Descending from the upper trace, the data show initial activity with 1.0 mM luminal Ca^2+^, activity after adding 20 µM 4,4′-DTDP to the cytoplasmic solution, activity following luminal perfusion with a 0.1 mM Ca^2+^ solution, then activity when luminal [Ca^2+^] was increased stepwise to 0.5 mM, 1 mM and 1.5 mM. *P*_o_ values for each recording are shown. (H) Mean *P_o_* before and after adding 20 µM 4,4′-DTDP (*n* = 10). (I–L) Mean data (*n* = 6–14) for (I) *P*_o_, (J) *T*_o_, (K) *T*_c_ and (L) *F*_o_. Error bars show ±s.e.m.; *P<0.05 (versus the value with 0.1 mM Ca^2+^).

Adding 1 mM DTT to the cytoplasmic solution did not cause a significant change in RyR2 activity (Fig. 2A,B), as reported previously (Hanna et al., 2011). This suggests that hyper-reactive cysteines that influenced *P*_o_ when luminal [Ca^2+^] was 1.0 mM were not significantly oxidised by ambient O_2_ (Eu et al., 2003). There was a trend towards an increase in mean *P*_o_ from 0.026±0.007 (±s.e.m.) to 0.039±0.01 when luminal Ca^2+^ was initially lowered to 0.1 mM. Subsequently, an unexpected and significant decline in *P*_o_ to ∼75% of the value recorded at 0.1 mM was observed as luminal [Ca^2+^] was increased to 1.5 mM (Fig. 2A,C). Notably, as in the absence of DTT, the change in *P*_o_ with increasing luminal [Ca^2+^] was mainly due to changes in the mean closed time and frequency of events, with a significant increase in *T*_c_ and a significant decrease in *F*_o_ but little change in mean open time (Fig. 2D–F).

### The effect of 20 µM 4,4′-DTDP in the cytoplasmic solution

We next examined the effect of adding 4,4′-DTDP to the cytoplasmic side of these ‘normal’ channels. It is well established that 10–20 µM 4,4′-DTDP increases RyR2 activity (Eager and Dulhunty, 1999; Eager et al., 1997; Marengo et al., 1998; Terentyev et al., 2008). However, to our knowledge, the effect of 4,4′-DTDP on the response of RyR2 to luminal [Ca^2+^] has been reported in only one previous study of RyR2 channels from normal dog heart, where 4,4′-DTDP abolished any difference between *P*_o_ at 0.02 mM and 2.0 mM luminal Ca^2+^ (Terentyev et al., 2008).

As expected, RyR2 activity increased significantly when 20 µM 4,4′-DTDP was added to the cytoplasmic solution with 1 mM luminal Ca^2+^ and 1 µM cytoplasmic Ca^2+^ (Fig. 2G,H). As in the absence of 4,4′-DTDP, *P*_o_ fell when luminal [Ca^2+^] was reduced to 0.1 mM. Then *P*_o_ increased significantly when luminal [Ca^2+^] was increased to 0.5 mM. No further increase in mean *P*_o_ was seen when [Ca^2+^] was increased to 1.0 or 1.5 mM, but values remained significantly higher than with 0.1 mM (Fig. 2G,I). Consistent with the results in the absence of 4,4′-DTDP, the increase in *P*_o_ as luminal [Ca^2+^] was increased in the presence of 4,4′-DTDP was due a significant decline in mean closed time and increase in the frequency of events, with little change in mean open time (Fig. 2J–L). It is notable that the mean *P*_o_ under most conditions is greater in the presence of 4,4′-DTDP (Fig. 2G–L) than in its absence (Fig. 1), owing to the shorter closed times and a higher frequency of events with 4,4′-DTDP. However, the relative increase in *P*_o_ (mean of relative changes calculated for individual channels) when luminal [Ca^2+^] was increased from 0.1 to 1.5 mM was not significantly different between that in the presence of 4,4′-DTDP (2.71±0.71-fold; ±s.e.m.) and its absence (4.25±1.2-fold).

### RyR2 response to luminal [Ca^2+^] in redox-buffered solutions

It is likely that DTT and 4,4′-DTDP added alone to the solutions produce changes in redox potential that are outside the physiological range of buffered redox potentials in intact cells. In addition, the DTT and 4,4′-DTDP situations are not equivalent in other respects. DTT is unlikely to cross the membrane at pH 7.4 (Hanna et al., 2011; Terentyev et al., 2008), so that the luminal side is either redox unregulated or oxidised owing to diffusion of ambient O_2_ into the solution (Eu et al., 2003). By contrast, as 4,4′-DTDP is lipid soluble, the luminal side of RyR2 would be exposed to 4,4′-DTDP after its addition to the cytoplasmic solution, so that both sides of RyR2 would experience extreme oxidising redox potentials.

**Fig. 3.**
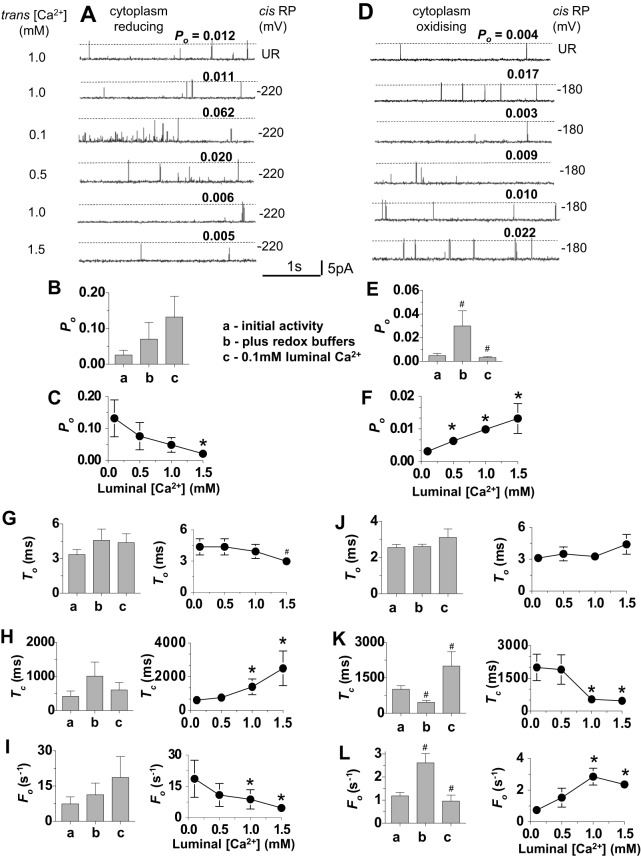
**Effect of cytoplasmic and luminal GSH∶GSSG redox buffers on the response of RyR2 channels to changes in luminal [Ca^2+^].** In this and subsequent figures, the redox potential (RP) in all luminal solutions was −180 mV, the cytoplasmic [Ca^2+^] was 1.0 µM, channel activity was recorded at +40 mV, channel opening is upwards and open probability (*P*_o_) values for each recording are shown above the broken line. (A–C,G–I) Channels exposed to cytoplasmic solutions having a reducing redox potential of −220 mV (*n* = 10). (D–F,J–L), channels exposed to cytoplasmic solutions having a more oxidised redox potential of −180 mV (*n* = 15). (A,D) Descending from the upper trace, the data show initial unbuffered redox (UR) activity with 1.0 mM luminal Ca^2+^, activity after addition of GSH∶GSSG with 1.0 µM luminal Ca^2+^, activity after perfusion with 0.1 mM Ca^2+^ luminal solution and replacement of GSH∶GSSG, then activity when luminal [Ca^2+^] was increased stepwise to 0.5 mM, 1 mM and 1.5 mM. *P*_o_ values for each recording are shown. (B,E) Mean *P*_o_ determined (a) for initial activity with 1 mM luminal Ca^2+^ and then (b) after adding GSH∶GSSG buffers and (c) after lowering luminal [Ca^2+^] to 0.1 mM. (C,F) Mean *P*_o_ after stepwise increases in luminal [Ca^2+^] from 0.1 mM to 1.5 mM. (G–L) Mean gating parameter values. (G,J) Mean open time (*T*_o_); (H,K) mean closed time, (*T*_c_); (I,L) mean frequency of opening (*F*_o_). The bar graphs show mean parameter values (a) for initial activity with 1 mM luminal Ca^2+^, (b) after adding GSH∶GSSG buffers and (c) after lowering luminal Ca^2+^ to 0.1 mM. The line graphs are plots of mean parameter values as a function of luminal [Ca^2+^]. Data are shown as the mean±s.e.m.; ^#^*P*<0.05 (versus the preceding condition); *P<0.05 (versus the mean value with 0.1 mM Ca^2+^).

To establish a more controlled physiological redox environment, GSH∶GSSG buffer systems were used to regulate the luminal and cytoplasmic redox potentials to values equivalent to those reported *in vivo* (Hwang et al., 1992). In these experiments, the luminal redox potential was kept constant at a relatively oxidised value of −180 mV. The cytoplasmic potential was set either at a more reduced level of −220 mV (Feng et al., 2000; Feng and Pessah, 2002; Jalilian et al., 2008), which is assumed to exist in healthy cells, or to a more oxidised value of −180 mV, which might exist in heart failure (Belevych et al., 2009). As observed with cytoplasmic DTT (Fig. 2), channel activity did not change significantly when the GSH∶GSSG buffers were added to achieve a reduced cytoplasmic redox potential (Fig. 3A,B). By contrast, channels were significantly activated when both the cytoplasm and lumen were buffered to an oxidising potential (Fig. 3D,E), as reported previously (Feng et al., 2000; Feng and Pessah, 2002; Jalilian et al., 2008). This increase in activity was reminiscent of that seen when 4,4′-DTDP was added to the cytoplasmic solution (Fig. 2).

There was a trend towards an increase in RyR2 activity when luminal [Ca^2+^] was lowered from 1.0 to 0.1 mM in the presence of the reducing cytoplasmic redox potential (Fig. 3A,B). When luminal Ca^2+^ was then increased stepwise, the activity of RyR2 fell, and mean *P*_o_ with 1.5 mM luminal Ca^2+^ was significantly lower than *P*_o_ with 0.1 mM Ca^2+^ (Fig. 3C). The reduction in *P*_o_ as luminal [Ca^2+^] was increased in the presence of a reducing redox potential was similar to that seen with cytoplasmic DTT (Fig. 2). Conversely, with the oxidising cytoplasmic redox potential, *P*_o_ fell significantly when luminal [Ca^2+^] was lowered from 1.0 to 0.1 mM (Fig. 3D,E) and then increased significantly as luminal [Ca^2+^] was increased (Fig. 3F). The increase in *P*_o_ with increasing luminal Ca^2+^ was similar to that seen under redox-unbuffered conditions (Fig. 1) or in the presence of 4,4′-DTDP (Fig. 2). It is also notable that, as with DTT or 4,4′-DTDP, the changes in channel activity with luminal [Ca^2+^] were due to alterations in the closed times and frequency of events, with minimal impact on open times (Fig. 3G–L). The results in Fig. 3 suggest that the changes in *P*_o_ with increasing luminal [Ca^2+^] are intrinsic responses of RyR2 channels under oxidising or reducing cytoplasmic redox conditions and are not an artefact of DTT, 4,4′-DTDP or the GSH∶GSSH buffer system.

### The effect of ATP on the RyR2 response to increasing luminal [Ca^2+^] with redox-buffered solutions

**Fig. 4.**
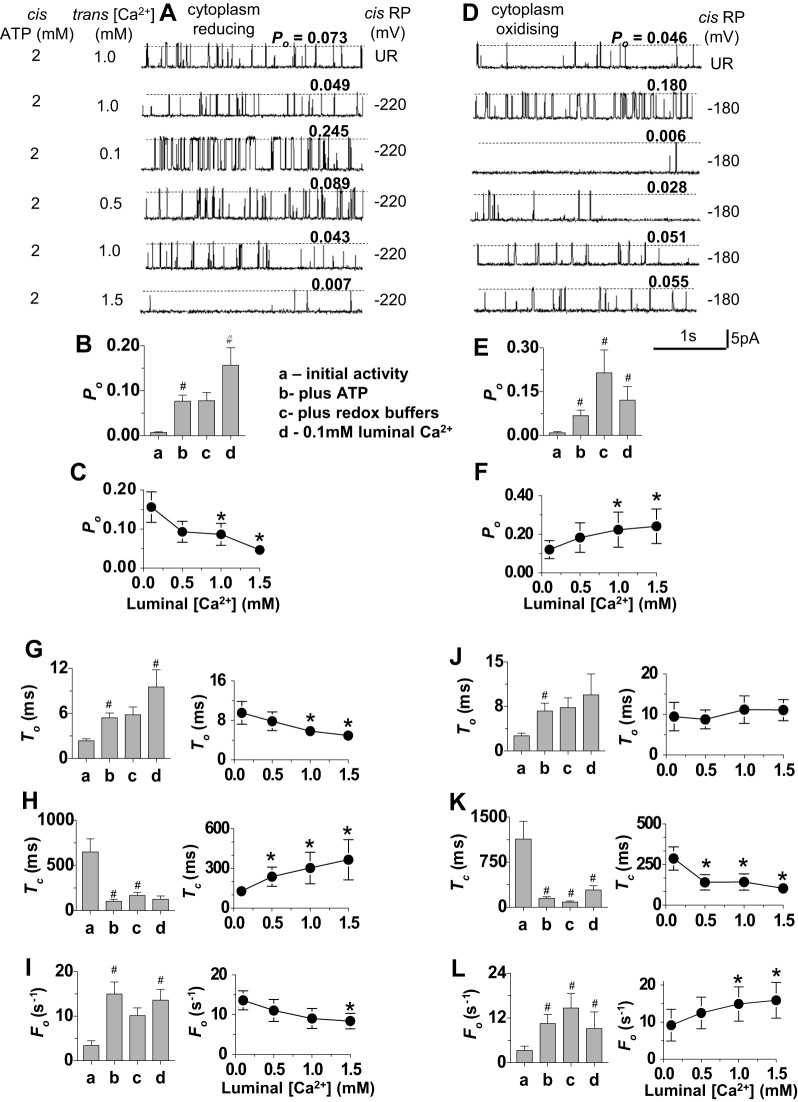
. **Redox-potential-dependent changes in channel activity are maintained in the presence of 2 mM ATP.** (A–C) Channels exposed to cytoplasmic solutions having a reducing redox potential (RP) of −220 mV (*n* = 16). (D–F) Channels exposed to cytoplasmic solutions having a more oxidised redox potential of −180 mV (*n* = 11). (A,D) Descending from the upper trace, the data show the initial unbuffered redox activity (UR) with 1.0 mM luminal Ca^2+^ and 2.0 mM Na_2_ATP in the cytoplasmic solution, the activity after addition of GSH∶GSSG buffers with 1.0 µM luminal Ca^2+^, the activity after perfusion with 0.1 mM Ca^2+^ luminal solution and replacement of GSH∶GSSG and then activity as the luminal [Ca^2+^] was increased stepwise to 0.5 mM, 1 mM and 1.5 mM. (B,E) Mean open probability (*P*_o_) determined (a) for initial activity with 1 mM luminal Ca^2+^, (b) after adding 2 mM Na_2_ATP, (c) after adding GSH∶GSSG buffers and (d) after lowering luminal [Ca^2+^] to 0.1 mM. (C,F) Mean data for *P*_o_ after stepwise increases in luminal [Ca^2+^]. (G–L) Mean gating parameter values. (G,J) Mean open time (*T*_o_); (H,K) mean closed time (*T*_c_); (I,L) mean frequency of opening (*F*_o_). The bar graphs show mean parameter values (a) for initial activity with 1 mM luminal Ca^2+^, (b) after adding 2 mM Na_2_ATP, (c) after adding GSH∶GSSG buffers and (d) after lowering luminal [Ca^2+^] to 0.1 mM. The line graphs are plots of mean parameter values as a function of luminal [Ca^2+^]. Data are shown as the mean±s.e.m.; ^#^*P*<0.05 (versus the preceding condition); *P<0.05 (versus the mean value with 0.1 mM Ca^2+^).

The experiments were then repeated in the presence of 2 mM cytoplasmic Na_2_ATP, as ATP is normally present in the myocyte cytoplasm and is a strong activator of RyR2 (Xu et al., 1996). RyR2 activity increased approximately fourfold to eightfold as expected when ATP was added (Fig. 4B,E). Apart from the generally higher *P*_o_, the effects of changes in luminal [Ca^2+^] were similar to those described above in the absence of ATP. The addition of redox buffers to create a reducing cytoplasmic environment did not alter RyR2 activity (Fig. 4A,B), whereas establishing a symmetrical oxidising environment increased channel activity (Fig. 4D,E). Lowering the luminal [Ca^2+^] to 0.1 mM caused a significant increase in *P*_o_ when the cytoplasmic redox potential was reducing (Fig. 4A,B), and a significant reduction in activity when the cytoplasmic redox potential was more oxidising (Fig. 4D,E). Subsequent increases in luminal [Ca^2+^] in steps from 0.1 to 1.5 mM were accompanied by a significant decline in *P*_o_ with the reduced cytoplasmic redox potential (Fig. 4A,C) or a significant increase with the more oxidising cytoplasmic redox potential (Fig. 4D,F).

As in the absence of ATP, the gating parameters were unaffected by the redox buffers when cytoplasmic redox potential was reducing. In contrast to the absence of ATP, the increase in *P*_o_ when luminal [Ca^2+^] was lowered from 1.0 mM to 0.1 mM was due to a significant increase in mean open time, in addition to the usual increase in opening frequency, and a trend towards a reduction in mean closed time (Fig. 4G–I). The subsequent decline in *P*_o_ with stepwise increases in luminal [Ca^2+^] was due to significant changes in all three parameters (Fig. 4G–I). Thus, channel open times were more subject to modulation by luminal [Ca^2+^] in the presence of cytoplasmic ATP with a reducing redox potential. Establishing symmetrical oxidising redox potentials again led to a significant increase in RyR2 activity, due to a reduction in the mean closed time and increase in the opening frequency with stepwise increases in luminal [Ca^2+^], without a change in the open times (Fig. 4J–L). In this oxidising cytoplasmic redox environment only mean closed time and opening frequency were modified by changes in luminal [Ca^2+^] (Fig. 4J–L).

### RyR2 responds to changes in luminal [Ca^2+^] in the presence of 1 mM free Mg^2+^ with redox-buffered solutions

**Fig. 5.**
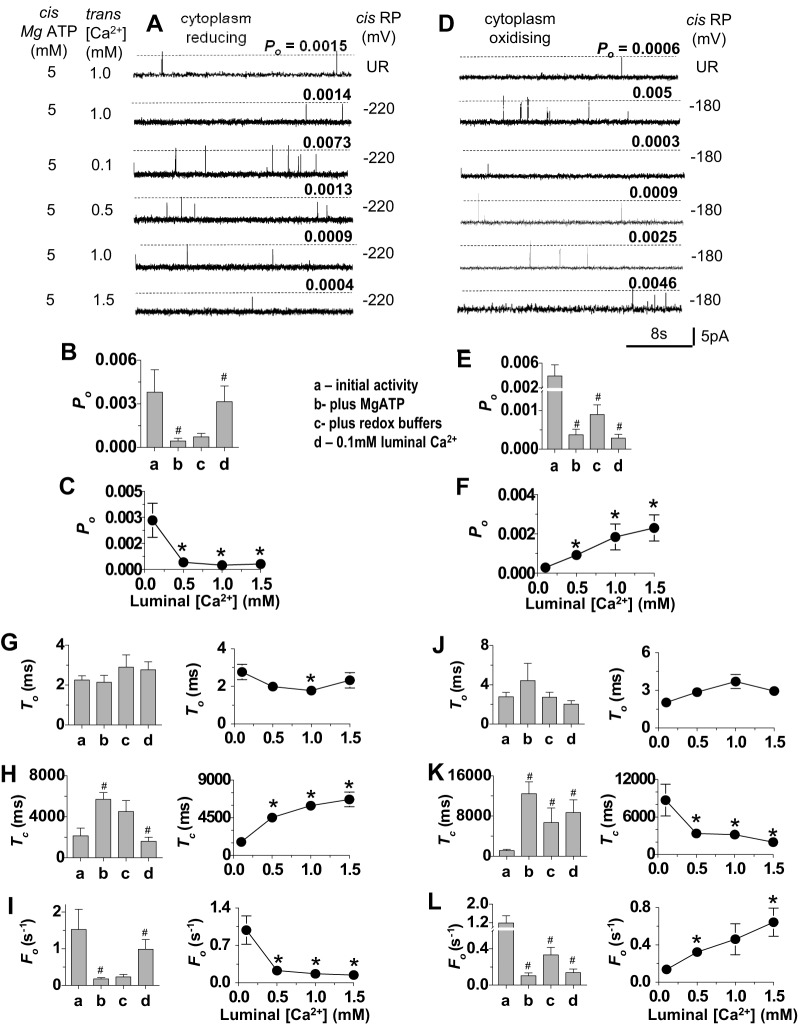
. **Redox-potential-dependent changes in channel activity are maintained in the presence of 1 mM free Mg^2+^.** (A–C) Channels exposed to cytoplasmic solutions with reducing redox potential (RP) of −220 mV (*n* = 8). (D–F) Channels exposed to cytoplasmic solutions with a more oxidised redox potential of −180 mV (*n* = 8). (A,D) Descending from the upper trace, the data show the initial unbuffered redox activity (UR) with 1.0 mM luminal Ca^2+^ and 5.0 mM MgATP in the cytoplasmic solution, the activity after addition of GSH∶GSSG buffers with 1.0 µM luminal Ca^2+^, the activity after perfusion with 0.1 mM Ca^2+^ luminal solution and replacement of GSH∶GSSG, and then the activity as luminal [Ca^2+^] was increased stepwise to 0.5 mM, 1 mM, and 1.5 mM. (B,E) Mean open probability (*P*_o_) determined (a) for initial activity with 1 mM luminal Ca^2+^, (b) adding 5.0 mM MgATP, (c) after adding GSH∶GSSG buffers and (d) after lowering luminal Ca^2+^ to 0.1 mM. (C,F) Mean data for *P*_o_ after stepwise increases in luminal [Ca^2+^]. (G–L) Mean gating parameter values. (G,J) Mean open time (*T*_o_); (H,K) mean closed time (*T*_c_); (I,L) mean frequency of opening (*F*_o_). The bar graphs show mean parameter values (a) for initial activity with 1 mM luminal Ca^2+^, (b) after adding 5.0 mM MgATP, (c) after adding GSH∶GSSG buffers and (d) after lowering luminal [Ca^2+^] to 0.1 mM. The line graphs are plots of mean parameter values as a function of luminal [Ca^2+^]. Data are shown as the mean±s.e.m.; ^#^*P*<0.05 (versus the preceding condition); *P<0.05 (versus the mean value with 0.1 mM Ca^2+^).

The experiments were finally performed in the presence of Mg^2+^ as well as ATP to more closely reflect the intracellular environment in the myocyte. Channel activity fell dramatically as expected with the addition of 5 mM MgATP (yielding 1 mM free Mg^2+^) (Fig. 5B,E). The length of the records in Fig. 5A,D is 24 s, rather than 3 s as in all earlier figures, in order to illustrate the sparse channel activity. In some channels only one or two brief openings occurred during 180 s of recording. Nevertheless, the usual significant increase in *P*_o_ was observed as luminal [Ca^2+^] was lowered from 1.0 mM to 0.1 mM when the cytoplasmic solution was buffered to the more reducing redox potential (Fig. 5A,B), and *P*_o_ then fell significantly as luminal [Ca^2+^] was increased in steps to 1.5 mM (Fig. 5A,C). Also, when the redox potential was more oxidising, *P*_o_ decreased in all eight cases when luminal [Ca^2+^] was reduced from 1.0 mM to 0.1 mM and then increased in each channel as luminal [Ca^2+^] rose to 1.5 mM (Fig. 5D–F). The changes in *P*_o_ were again due to changes in mean closed times and frequency of opening, with no consistent effect on the open times (Fig. 5G–L).

Therefore, we observed characteristic changes in RyR2 activity with increasing luminal [Ca^2+^] when the redox potential was buffered with GSH∶GSSG in the presence of 1 mM Mg^2+^. The changes in channel gating are consistent with those seen under all other conditions, although occurring over a much lower range of *P*_o_ values that might, in fact, approach levels of RyR2 activity in myocytes from normal hearts during diastole, when channel activity is likely to be relatively low.

We explored the possibility that a higher cytoplasmic [Ca^2+^] would influence the RyR2 response to luminal [Ca^2+^] under reducing cytoplasmic redox conditions in the presence of 5 mM MgATP. The mean *P*_o_ values at each stage of the experiment were at least two orders of magnitude higher than with 1 µM cytoplasmic Ca^2+^. However, the changes in *P*_o_ at each step with 10 µM cytoplasmic Ca^2+^ (Table 1) were similar to changes seen with 1 µM Ca^2+^ (Fig. 5). *P*_o_ fell in all channels when 5 mM MgATP was added, did not change consistently with the addition of redox buffers, increased when luminal [Ca^2+^] was reduced to 0.1 mM and then fell with increases in luminal [Ca^2+^] to 1.0 mM and 1.5 mM. A tenfold variability in *P*_o_ between individual channels was similar to that at lower cytoplasmic Ca^2+^ (compare s.e.m. in Table 1 and in Figs 1–5).

**Table 1.**
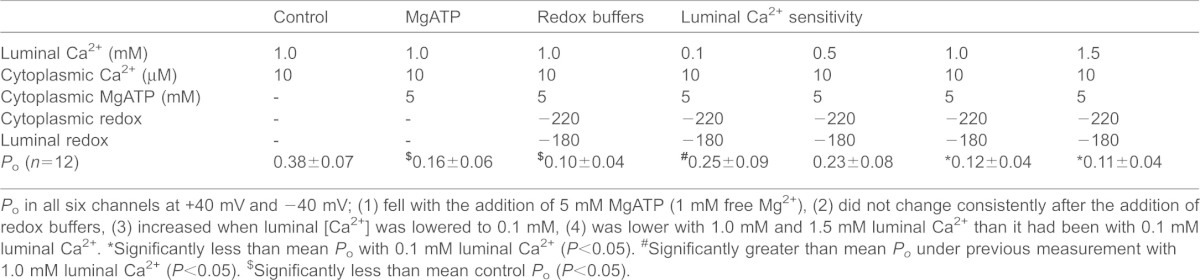
Luminal Ca^2+^ sensitivity in the presence of 10 µM cytoplasmic Ca^2+^ with 5 µM MgATP and reducing cytoplasmic redox potential

*P*_o_ in all six channels at +40 mV and −40 mV; (1) fell with the addition of 5 mM MgATP (1 mM free Mg^2+^), (2) did not change consistently after the addition of redox buffers, (3) increased when luminal [Ca^2+^] was lowered to 0.1 mM, (4) was lower with 1.0 mM and 1.5 mM luminal Ca^2+^ than it had been with 0.1 mM luminal Ca^2+^.

* Significantly less than mean *P*_o_ with 0.1 mM luminal Ca^2+^ (*P*<0.05).

# Significantly greater than mean *P_o_* under previous measurement with 1.0 mM luminal Ca^2+^ (*P*<0.05).

$ Significantly less than mean control *P*_o_ (*P*<0.05).

## DISCUSSION

We have examined the effect of redox potential on the response of single RyR2 channels in lipid bilayers to changes in luminal [Ca^2+^] within the physiological range experienced in the sarcoplasmic reticulum. The experiments focused on the increase in luminal [Ca^2+^] that would occur during diastole, as Ca^2+^ is pumped from the cytoplasm into the sarcoplasmic reticulum. We show a novel response of RyR2 open probability to increases in luminal [Ca^2+^] from 0.1 mM to 1.5 mM under reducing cytoplasmic redox conditions. This robust decline in activity of ∼75% was revealed when the cytoplasmic side of the channel was exposed to 2 mM DTT or when the redox potential in the cytoplasmic solution was buffered with GSH∶GSSG to −220 mV. The inhibitory effect with increasing luminal [Ca^2+^] was seen when channels were partially activated by 1 µM cytoplasmic Ca^2+^ alone, with 1 µM cytoplasmic Ca^2+^ plus 2 mM Na_2_ATP or when channels were strongly inhibited in the presence of 1 mM free Mg^2+^. In contrast to the decline in activity under reducing redox conditions, channel activity increased with increasing [Ca^2+^] in the absence of redox reagents, as has previously been reported using the same range of [Ca^2+^] (Dulhunty et al., 2012; Györke et al., 2004; Qin et al., 2008; Sitsapesan and Williams, 1994). A threefold to fivefold increase in activity was observed when the cytoplasmic redox potential was experimentally unregulated, unphysiologically oxidising (20 µM 4,4′-DTDP) or when both the cytoplasmic and luminal solutions were buffered with GSH∶GSSG to an oxidising redox potential of −180 mV. Once again, this response was seen in the absence or presence of 2 mM Na_2_ATP or with 1 mM free Mg^2+^ and ATP. It is notable that, under most conditions, variations in sensitivity to luminal [Ca^2+^] were primarily dependent on changes in the time spent in the closed state and, hence, the frequency of events, rather than changes in the duration of open events. This specific action in modulating channel closed times is indicative of an effect of [Ca^2+^] on the luminal [Ca^2+^] sensor, rather than feed-through activation where luminal Ca^2+^ ions flow through the channel to bind to cytoplasmic Ca^2+^ regulatory sites and produce long channel openings (Laver, 2007).

### RyR2 channel gating under control conditions

It is recognised that channel gating varies between individual RyR channels independently of cytoplasmic [Ca^2+^] from 0.1 to 1000 µM (Copello et al., 1997; Laver, 2007; Marengo et al., 1998). The variability is likely due to the many regulatory sites in the four subunits of the protein, which form the largest known ion channel. It is also likely that not all regulatory residues that are subject to oxidation, glutathionylation, nitrosylation, phosphorylation, etc are covalently complexed at any one time *in vivo* and, hence, in native RyR channels incorporated into the bilayer. As a result, the *P*_o_ for individual channels can vary over a wide range, and average control *P*_o_ in any data set can also vary depending on the number of high activity and low activity channels in the data set. What is more important than the *P*_o_ values is whether the channels respond in the same way to experimental challenges. This was the case in the present experiments, where cytoplasmic addition of ATP increased *P*_o_ in all channels, MgATP reduced *P*_o_ in all channels and 4,4′-DTDP or a cytoplasmic redox potential of −180 mV increased *P*_o_ in all channels. In the majority of channels, *P*_o_ increased when luminal [Ca^2+^] was lowered to 0.1 mM under reducing conditions and fell as luminal [Ca^2+^] was subsequently increased. Conversely, under oxidising conditions, activity fell in the majority of channels when luminal Ca^2+^ was lowered to 0.1 mM and then increased as luminal Ca^2+^ was subsequently increased. Changes in activity that were not significant according to a Student's *t*-test, were significant (*P*<0.05) according to a sign test, because parameters in a critical number of individual channels changed in the same direction (either increased or decreased) with the particular treatment.

Our aim was to replicate diastolic conditions as closely as possible; however, the experiments were conducted with a cytoplasmic Ca^2+^ of 1 µM, rather than lower-end diastolic concentrations of 100–300 nM. This was done in order to see quantifiable activity, especially in the presence of 1 mM Mg^2+^, where activity of individual channels is too low to measure if cytoplasmic Ca^2+^ is <1 µM (Laver, 2007). However, data obtained with 1 mM cytoplasmic Mg^2+^ and 1 µM cytoplasmic Ca^2+^ can be extrapolated to lower cytoplasmic [Ca^2+^] (Chen et al., 2013; Laver, 2007). With higher cytoplasmic [Ca^2+^] (10 µM), reducing cytoplasmic redox potential, Mg^2+^ and ATP, there were the same changes in *P*_o_ as a function of luminal [Ca^2+^] (Table 1) as seen with 1 µM Ca^2+^ (Fig. 5), but relative changes in *P*_o_ were larger with 1 µM Ca^2+^. Greater relative changes in *P*_o_ in response to changes in luminal [Ca^2+^] at lower cytoplasmic [Ca^2+^] are also reported in the absence of redox buffers (Lukyanenko et al., 1996).

### Implications for the redox potential in the microdomain surrounding the cytosolic surface of RyR2

The redox potential in the cytoplasm of healthy myocytes is considered to be more reduced than the redox potential inside the sarcoplasmic reticulum, with a cytosolic GSH∶GSSG ratio between 30∶1 and 100∶1 (Hwang et al., 1992). However, our results, taken together with measurements of Ca^2+^ release from the sarcoplasmic reticulum or normal cardiac myocytes (Shannon et al., 2002), suggest that the redox potential in the microdomain surrounding the cytoplasmic side of RyR2 is not reducing. An increase in Ca^2+^ leak is seen as luminal [Ca^2+^] increases in healthy myocytes (Lukyanenko et al., 1996; Lukyanenko et al., 2001; Satoh et al., 1997; Shannon et al., 2003). It is likely that this increase in leak is caused by an increase in RyR2 open probability, as is seen in bilayer experiments under redox-unregulated conditions (Dulhunty et al., 2012; Györke et al., 2004; Qin et al., 2008; Sitsapesan and Williams, 1994). We see a similar increase as luminal [Ca^2+^] is increased from 0.1 to 1.5 mM in the presence of 20 µM 4,4′-DTDP (Fig. 2) or with an oxidising cytoplasmic redox potential of −180 mV (Figs 3–5). The consistent decline in RyR2 open probability when the cytoplasmic redox potential is reducing is at odds with the observations of enhanced diastolic Ca^2+^ release with increasing Ca^2+^ load (Shannon et al., 2002). Therefore, the results suggest that the redox potential in the junctional microdomain surrounding the cytosolic side of RyR2 might, in fact, be more oxidising than the bulk of the cytoplasm.

**Fig. 6.**
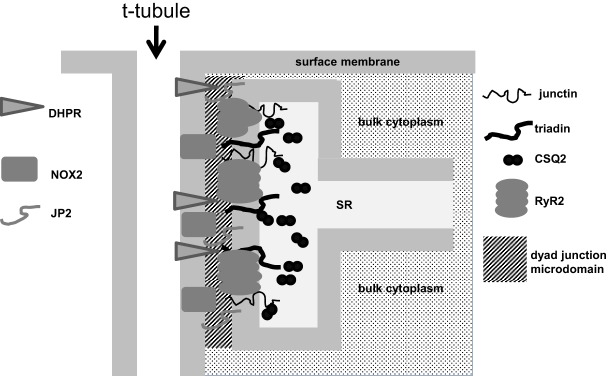
. **Illustration of the likely cytoplasmic microdomain within the junction between the T-tubule and the sarcoplasmic reticulum in a cardiac myocyte.** The width of the junction is similar to the width of the transverse (T)-tubule and sarcoplasmic reticulum (SR) membranes that form its borders. The cleft contains cytoplasmic domains of the DHPR and NOX2 embedded in the T-tubule membrane and JP2, RyR2, triadin and junctin embedded in the sarcoplasmic reticulum membrane, in addition to many other identified junctional components that are not shown.

It would not be surprising if the tiny (∼10 nM) junctional gap between the transverse (T)-tubule and the sarcoplasmic reticulum membrane forms a microdomain within the cytoplasm. Not only is the gap narrow (∼10 nm) but it is crowded with many junctional proteins, including the huge cytoplasmic domain of RyR2, which effectively forms a two-dimensional crystal lattice (Yin et al., 2005), the dihydropyridine receptor (DHPR) α and β subunits (Bers, 2002), junctophilin 2 (JP2), (Zhang et al., 2014), the NADPH oxidase NOX2 (Donoso et al., 2014) (Fig. 6) and other proteins too numerous to mention. An oxidising redox potential within this junctional microdomain could be maintained by NOX2 in the T-tubule membrane that is colocalised with RyR2 (Sánchez et al., 2005). Activation of NOX2 increases ROS production, and ROS oxidise RyR2, which might, in turn, increase the sensitivity of RyR2 to activating factors (Prosser et al., 2011). This response is essential in ‘tuning’ normal cardiac function and is cardioprotective in healthy myocytes (Donoso et al., 2014; Prosser et al., 2011; Rosc-Schlüter et al., 2012; Sánchez et al., 2008; Zhang et al., 2013). However, the response can precipitate arrhythmia under pathological conditions (Prosser et al., 2011; Zhang et al., 2013).

Our hypothesis is that the junctional microdomain is maintained at a mildly oxidising redox potential under healthy conditions and that this is essential for the normal increase in RyR2 activity as luminal [Ca^2+^] increases during diastole. However, the situation becomes detrimental if RyR2 is hypersensitive to activation in disorders such as catecholaminergic polymorphic ventricular tachycardia or if the redox potential is further increased and the degree of thiol modification is increased with NOX2 stimulation and excess ROS production as a result of chronic β adrenergic stimulation, as in heart failure (Sánchez et al., 2005; Zhang et al., 2013). As we did not measure the dyad redox potential, an alternative hypothesis is that the junction is normally at a reduced redox potential owing to the large cytosolic redox buffer capacity, and that RyR2 is, in fact, inhibited during diastolic sarcoplasmic reticulum Ca^2+^ loading. This would increase the concentration of stored Ca^2+^ and decrease *P*_o_ and the possibility of spontaneous Ca^2+^ release. However, the fact that all measures of sarcoplasmic reticulum Ca^2+^ leak indicate an increase in leak with Ca^2+^ overload suggests that this is not the case (Fauconnier et al., 2011; Marks, 2013; Pogwizd and Bers, 2004; Shannon et al., 2002; Tian et al., 2013; Zahradníková et al., 2010).

It is notable that, although *P*_o_ values were higher in the presence of oxidising redox reagents than in their absence, the relative activation of RyR2 as luminal [Ca^2+^] was raised from 0.1 to 1.5 mM did not vary substantially with the extent of oxidation and was similar with 4,4′-DTDP, with oxidising GSH∶GSSG buffer or indeed with no added redox reagents. Thus, in the absence of redox buffers, *P*_o_ was minimal at 0.1 mM luminal Ca^2+^ and greater at higher concentrations, but *P*_o_ was higher at each luminal [Ca^2+^] in the presence of the oxidising redox agents. If this reflected the situation in the failing heart under oxidative stress, the threshold *P*_o_ for spontaneous Ca^2+^ release would be reached at lower luminal [Ca^2+^] in the thiol-modified failing RyR2, and this would lead to a higher cytoplasmic [Ca^2+^], stimulation of NCX activity, DADs and arrhythmia (Bers, 2002). Clearly, many more factors are present and impact on RyR2 in intact cardiac myocytes than can be included in bilayer solutions. Therefore, the *P*_o_ values that we see in the bilayer situation might not be identical to the channel open probability *in vivo*, although values with 5 mM MgATP (1 mM free Mg^2+^ and ATP) are likely closest to the *in vivo* values. Overall, our data reveal a basic response of RyR2 channels to changes in luminal [Ca^2+^] that are consistent with changes in Ca^2+^ release in intact cells, with the notable exception of the responses under reducing redox conditions. The data reveal a baseline response that can be modulated up or down by other endogenous RyR2 regulators.

### Molecular mechanisms and physiological significance of the paradoxical response of RyR2 to luminal Ca^2+^

The redox-dependence of RyR2 responses to changes in luminal [Ca^2+^] suggest that redox potential alters the inter- or intra-molecular factors that link the luminal [Ca^2+^] sensor or sensors to the channel-gating mechanism. This would not be surprising and is consistent with the established influence of thiol modification on channel gating (Eager and Dulhunty, 1999; Eager et al., 1997; Marengo et al., 1998; Terentyev et al., 2008; Donoso et al., 2014). The question is whether RyR2 is ever exposed to a reducing cytosolic environment and what the consequences would be for cardiac function. It has been suggested that a basal level of NOX2 activity is required for normal heart function and that suppression of NOX2 can induce cardiac injury (Zhang et al., 2013; Donoso et al., 2014), a concept encompassed in the title “NADPH oxidases in heart failure: poachers or gamekeepers?” (Zhang et al., 2013). This raises the possibility that a part of the protective effect of NOX2 is in maintaining a mildly oxidised redox potential in the dyad junction. If the redox potential became more reducing, and RyR2 was sufficiently inhibited, the sarcoplasmic reticulum might fill more rapidly and reach a threshold for detrimental spontaneous Ca^2+^ release within the diastolic interval, and less Ca^2+^ would be available for release during systole. Because neither scenario is observed as a function of sarcoplasmic reticulum Ca^2+^ load under normal conditions, it seems unlikely that the environment is reducing and that *P*_o_ decreases with sarcoplasmic reticulum refilling. From a different perspective, a population of RyR2 channels are located in longitudinal sarcoplasmic reticulum and are exposed to the bulk cytoplasmic redox potential, which might be more reduced than the junctional potential. If this is the case, then the overall Ca^2+^ fluxes across the sarcoplasmic reticulum membrane would be a complex combination of the responses of the different RyR2 populations to changes in luminal Ca^2+^.

### RyR2 response to changes in luminal [Ca^2+^] in the absence of added redox reagents

The similar response of RyR2 to increases in luminal [Ca^2+^] in the absence of added redox reagents and in the presence of oxidising agents is curious and suggests that there might be an intrinsic oxidising redox potential in the bilayer solutions. Indeed, both cytoplasmic and luminal solutions might, in fact, contain oxidising factors in the absence of added redox reagents, owing to ambient O_2_ dissolved in the solutions. RyR2 thiol content is reduced and [^3^H]ryanodine binding is enhanced (indicating increased channel activity) at ambient partial O_2_ pressure (pO_2_, 150 mm Hg) (Sun et al., 2008). In addition, O_2_ in solution can interact with NOX enzymes that might be associated with the sarcoplasmic reticulum vesicles (Eu et al., 2003). ROS, such as hydrogen peroxide (H_2_O_2_), are generated in proportion to pO_2_ by NADPH oxidase 4 (NOX4) in the sarcoplasmic reticulum membrane. H_2_O_2_ is responsible for pO_2_-dependent RyR1 oxidation (Sun et al., 2011). Therefore, O_2_ could activate NOX4 in the sarcoplasmic reticulum vesicle membrane incorporated into the bilayer or associated with unincorporated sarcoplasmic reticulum vesicles remaining in the cis solution (Lassègue et al., 2012). Although sarcoplasmic reticulum vesicles are present in only small quantities, they might generate sufficient H_2_O_2_ to increase the oxidising redox potential in the bilayer solutions in the absence of any added redox reagents. Taken together, our data are consistent with the notion that the redox potential of solutions bathing lipid bilayers are intrinsically oxidising.

### Maintained sensitivity to luminal [Ca^2+^] in the presence of 4,4′-DTDP and with 1 mM free cytosolic Mg^2+^

In contrast to our results, it has been reported that the luminal Ca^2+^ sensitivity of normal canine RyR2 channels at 0.02 and 2.0 mM is lost after exposure to 4,4′-DTDP (Terentyev et al., 2008). The findings are difficult to reconcile with measurements in intact cardiac myocytes showing an increase in Ca^2+^ leak through RyR2 when sarcoplasmic reticulum load increases, indicating an increase in *P*_o_ as luminal [Ca^2+^] increases (Lukyanenko et al., 1996; Lukyanenko et al., 2001; Satoh et al., 1997; Shannon et al., 2003). Our work was limited to physiological luminal [Ca^2+^] of 0.1 to 1.5 mM, but changes in this range should have been encapsulated within the broader range of 0.02 to 2.0 mM. The reasons for the differences are not clear and should be explored further in the future. However, possibilities include species differences (dog versus sheep) and also the association status of cardiac calsequestrin (CSQ2) and junctin. Both CSQ2 and junctin regulate RyR2 sensitivity to luminal [Ca^2+^] (Altschafl et al., 2011; Chen et al., 2013; Dulhunty et al., 2012; Györke et al., 2004; Knollmann et al., 2006; Qin et al., 2008), although the nature and function of the regulation remains to be elucidated. We ensured that CSQ2 remained associated with RyR2 by limiting exposure to 0.1 mM luminal Ca^2+^ and not exposing channels to more than 1.5 mM luminal Ca^2+^ (Wei et al., 2006).

The response of RyR2 to increasing luminal [Ca^2+^] between 0.1 and 1.5 mM is maintained in the presence of 1 mM free Mg^2+^ and ATP at concentrations found in normal intact myocytes. It is clear that, in the presence of an oxidised cytoplasmic environment, Ca^2+^ release from the sarcoplasmic reticulum increases with sarcoplasmic reticulum Ca^2+^ load in a manner consistent with increasing RyR2 *P*_o_ in myocytes (Shannon et al., 2002). By contrast, it has been reported that RyR2 sensitivity to luminal [Ca^2+^] is lost in the presence of 1 mM free cytoplasmic Mg^2+^ and ATP in the absence of added redox buffers (Chen et al., 2013). It is possible that the sensitivity to luminal [Ca^2+^] in the presence of 1 mM free cytoplasmic Mg^2+^ and ATP also requires the presence of GSH∶GSSG and potentially other cellular redox buffers. However, the many other differences in the preparation of sarcoplasmic reticulum and in the ionic composition of the bilayer solutions might also account for the different results.

### Conclusions

Here, we show for the first time that an oxidising cytosolic environment is a prerequisite for the increase in RyR2 open probability that is associated with the increase in luminal [Ca^2+^] as the sarcoplasmic reticulum refills during diastole. If the redox potential in the cytoplasm is reducing there is a robust decline in RyR2 open probability as the [Ca^2+^] in the sarcoplasmic reticulum is increased through the diastolic range. The results imply that the redox potential in the junctional microenvironment surrounding the cytoplasmic surface of RyR2 is normally oxidising, consistent with an oxidative influence of NOX2 in the T-tubule membrane. Overall, the results highlight the likely existence of microdomains within the cytoplasm that allow conditions to differ significantly from those in the bulk cytoplasm.

## MATERIALS AND METHODS

### Materials

Phospholipids were from Avanti Polar Lipids (Alabaster, AL). All other chemicals were obtained from Sigma-Aldrich (Castle Hill, NSW, Australia).

### Preparation of sarcoplasmic reticulum vesicles

Sarcoplasmic reticulum vesicles were prepared from sheep heart as described previously (Eager and Dulhunty, 1999).

### Single channel activity

Sarcoplasmic reticulum vesicles were incorporated into artificial planar bilayers as described previously (Beard et al., 2002; Eager and Dulhunty, 1999; Hanna et al., 2011), with the cytoplasmic (cis) solution containing 230 mM caesium methanesulfonate (CsMS), 20 mM CsCl, 1 mM CaCl_2_ and 10 mM tetraethylsulfamide (TES) pH 7.4, and the luminal (trans) solution containing 30 mM CsMS, 20 mM CsCl, 1 mM CaCl_2_ and 10 mM TES pH 7.4. Sarcoplasmic reticulum vesicles (∼50 µg) were added to the cis solution so that the cytoplasmic surface of the sarcoplasmic reticulum and RyR2 faced the cis solution after incorporation into the lipid bilayer. After channel incorporation, free cis Ca^2+^ was decreased to 1 µM with the addition of ∼1.32 mM BAPTA, and 200 mM CsMS was added to the trans solution to achieve symmetrical [Cs^+^]. Luminal [Ca^2+^] was reduced from 1 mM to 0.1 mM by perfusion, and was then increased in three steps to 1.5 mM by the addition of CaCl_2_. Free [Ca^2+^] in all solutions was determined using a Ca^2+^-selective electrode (Radiometer Analytical, Villeurbanne Cedex, France).

### Single channel recording and analysis

The techniques have been described previously (Beard et al., 2002; Eager and Dulhunty, 1999; Hanna et al., 2011). By convention, electrical potentials are expressed as *V*_cis_−*V*_trans_. All experiments were performed at 23±2°C. Single-channel parameters were obtained using the Channel 2 programme (developed by Peter W. Gage and Michael Smith, John Curtin School of Medical Research, Canberra, ACT, Australia) applied to 60 to 90 s of channel activity. The threshold levels for channel opening were set to exclude baseline noise at ∼20% of the maximum single-channel conductance and open probability (*P*_o_), mean open time (*T*_o_), mean closed time (*T*_c_) and open frequency (*F*_o_) were measured. Data obtained at +40 mV and −40 mV were analysed separately. No voltage-dependent differences were evident and so data for +40 mV and −40 mV were combined in the final mean values presented in the Results.

### Redox buffering

The redox potential was manipulated with a GSH∶GSSG buffer (Feng et al., 2000; Feng and Pessah, 2002; Jalilian et al., 2008). An oxidising redox potential of −180 mV was established with 0.1 mM GSH and 0.95 mM GSSG. A reducing redox potential of −220 mV was achieved with 4.0 mM GSH plus 0.072 mM GSSG. GSH and GSSG were freshly prepared and added individually in appropriate amounts to the cis and trans solutions.
