# Correction: Cardiac ryanodine receptor activation by a high Ca^2+^ store load is reversed in a reducing cytoplasmic redox environment

**DOI:** 10.1242/jcs.198705

**Published:** 2016-11-15

**Authors:** Amy D. Hanna, Alex Lam, Chris Thekkedam, Esther M. Gallant, Nicole A. Beard, Angela F. Dulhunty

There was an error published in *J. Cell Sci.* **127**, 4531-4541.

Incorrect concentrations of the components used to establish the oxidising redox potential were given in the ‘Redox buffering’ section of the Materials and Methods. The correct sentence should read: An oxidising redox potential of −180 mV was established with 0.95 mM GSH and 0.1 mM GSSG.

The authors apologise to the readers for any confusion that this error might have caused.

